# Estimation of patient setup uncertainty using BrainLAB Exatrac X‐Ray 6D system in image‐guided radiotherapy

**DOI:** 10.1120/jacmp.v16i2.5102

**Published:** 2015-03-08

**Authors:** Erminia Infusino, Lucio Trodella, Sara Ramella, Rolando M. D'Angelillo, Carlo Greco, Aurelia Iurato, Luca E. Trodella, Alessandro Nacca, Patrizia Cornacchione, Alessandra Mameli

**Affiliations:** ^1^ Department of Radiotherapy Campus Bio‐Medico University of Rome Rome Italy

**Keywords:** setup errors, image‐guided radiotherapy, X‐ray images, infrared system, treatment planning

## Abstract

The purpose of this study was to evaluate setup uncertainties for brain sites with ExacTrac X‐Ray 6D system and to provide optimal margin guidelines. Fifteen patients with brain tumor were included in this study. Two X‐ray images with ExacTrac X‐Ray 6D system were used to verify patient position and tumor target localization before each treatment. The 6D fusion software first generates various sets of DRRs with position variations in both three translational and three rotational directions (six degrees of freedom) for the CT images. Setup variations (translation and rotation) after correction were recorded and corrected before treatment. The 3D deviations are expressed as mean±standard deviation. The random error $\left(\Sigma (\sigma_{i})\right)$, systematic error $\left(\mu_{i}\right)$, and group systematic error $\left(M(\mu_{i})\right)$ for the different X‐ray were calculated using the definitions of van Herk.^(1)^ Mean setup errors were calculated from X‐ray images acquired after all fractions. There is moderate patient‐to‐patient variation in the vertical direction and small variations in systematic errors and magnitudes of random errors are smaller. The global systematic errors were measured to be less than 2.0 mm in each direction. Random component of all patients are smaller ranging from 0.1–0.3 mm small. The safety margin (SM) to the lateral, is 0.5 mm and 2.6 mm for van Herk^(1)^ and Stroom et al.,^(2)^ respectively, craniocaudal axis is 1.5 mm and 3.4 mm, respectively, and with respect to the antero–posterior axis, 2.3 mm and 3.9 mm. Daily X‐ray imaging is essential to compare and assess the accuracy of treatment delivery to different anatomical locations.

PACS number: 87.55.D

## I. INTRODUCTION

The radiotherapy process includes many uncertainties that should be considered in the planning target volume definition in order to avoid any geometrical lack during treatment delivery.

Setup errors should be considered. Patient setup error can be defined as the difference between the actual and the planned position of the patient with respect to the treatment beams during irradiation.

These uncertainties can be also separated in systematic and random components. The systematic component can be defined as a patient‐dependent average displacement between treatment anatomy and planning scan anatomy. It is mainly due to the fact that the reference geometry of the planning CT scan is actually a capture of an arbitrary random displacement. The random component, on the other side, can be defined as the fluctuation over the time around the systematic displacement.^(3)^ The distinction between a systematic and a random component reflects a different impact on treatment dose. In fact, it has been demonstrated that, to ensure dose homogeneity coverage for target volume, systematic errors require margins three to four times as large as comparable random displacements.^4^, ^5^

Stereotactic brain of intracranial lesions requires maximum accuracy of treatment planning and delivery to ensure that the irradiation doses are confined precisely to the target structures. Modern radiotherapy techniques provide dose distributions that conform to target volumes while avoiding neighboring normal tissues. Increasingly conformal and complex treatment plans have steep dose gradients, which increases the need for more accurate setup and delivery. Image guidance systems can improve the ability to accurately deliver highly conformal dose distributions in applications in which setup uncertainty or organ motion is high.^(6)^

Image guidance plays an important role in stereotactic brain because it supports accurate target localization and avoidance of adjacent organs at risks (OAR). A number of imaging modalities, including ultrasound, video imaging, two‐dimensional radiographic imaging (kV and MV), computed tomography (CT) (conventional CT as well as kV and MV cone‐beam CT), and magnetic resonance imaging (MRI), are used in image‐guided radiotherapy (IGRT). Several IGRT systems are now commercially available and have been successfully implemented for clinical applications. The BrainLAB ExacTrac X‐Ray 6D stereotactic IGRT system uses a combination of optical positioning and kV radiographic imaging to accurately position patients and make online positioning corrections. It has been clinically used for intracranial and extracranial radiosurgery with success.^7^, ^8^ This study analyzes patient positioning corrections that are performed during treatments using an ExacTrac X‐Ray 6D system, that is mainly an integration of two subsystems: an infrared (IR)‐based optical positioning system (ExacTrac) for initial patient setup and precise control of couch movement, and a radiographic kV X‐ray imaging system (X‐Ray 6D) for position verification and readjustment. The position verification was done before any treatment fields and readjusted during treatments.

The purpose of this study is to assess the use of daily pretreatment imaging for brain disease sites by quantifying setup errors treated with stereotactic radiotherapy at our institution.

## II. MATERIALS AND METHODS

### A. System description

**Figure 1:**
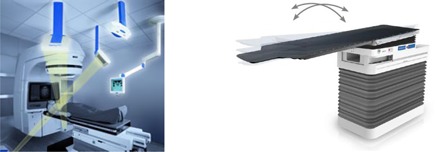
The body image‐guided system showing oblique configurations of the X‐ray imaging devices.

The infrared tracking component of the ExacTrac X‐Ray 6D system (BrainLAB AG, Feldkirchen, Germany) includes two IR cameras, passive IR reflecting spheres placed on a patient's surface (Fig. 1). The IR cameras are rigidly mounted to a metal bar attached to the ceiling and emit a low IR signal that is reflected and analyzed for positioning information. The camera is made up of two IR cameras and one video camera in the center of the system. The two IR cameras detect reflective markers, while the video camera provides patient verification. A two‐step calibration procedure has been established to ensure that the IR cameras can accurately determine the position of IR reflectors in the treatment room. The first step corrects for distortions in the IR system and creates a coordinate space, while the second step provides the system with the location of the linear accelerator isocenter. Studies by Wang et al.^(9)^ have demonstrated that the position of each IR‐reflecting sphere can be determined to less than 0.3 mm. Automatic setup can then be easily achieved by moving the couch to match the marker's position with those recorded in a CT image. In addition, the software also provides rotational offsets along three primary axes. However, the external markers have to be positioned in a relatively stable location to achieve accurate setup.

#### A.1 X‐ray

The X‐ray component consists of two floor‐mounted kV X‐ray tubes, projecting medial, anterior, and inferior obliquely into two corresponding flat panel detectors mounted on the ceiling (Fig. 1). The configuration of this X‐ray system is unique compared to the general diagnostic X‐ray systems in that the X‐ray tubes and corresponding detector panels are in fixed positions, the X‐rays project in an oblique direction relating to the patients, and the source isocenter and source detector distance is relatively large. Two X‐ray images are obtained after a patient is initially set up with the ExacTrac (infrared) system. The 6D fusion software first generates various sets of DRRs with position variations in both three translational and three rotational directions (six degrees of freedom) from the planning CT images. It then compares these DRRs with the corresponding X‐ray images and obtains the set of DRRs with the maximal similarity to the corresponding X‐ray images. The best match is thus determined and the three translational and three rotational position variations used to generate the set of DRRs are the 6D offsets to fuse the images.

### B. Treatment system and planning technique

Between March 2014 and October 2014, a total of 15 patients were treated with cerebral metastases with stereotactic radiotherapy at our institution, each with a maximum diameter of no more than 4 cm on contrast‐enhanced MRI scans. The primary involved organs were lung (70%) and breast (30%). The prescribed dose for stereotactic radiation therapy (SRT) usually was 30 Gy in 3 fractions, the dose was prescribed to the isodose line covering the PTV (generally the 65% isodose line). A planning target volume (PTV) was generated based upon a 3 mm symmetric expansion of the primary CTV.

The patients were treated on a Varian TrueBeam treatment machine (Varian Medical Systems, Palo Alto, CA) using RapidArc technology.

For this study, all target definition and dynamic conformal arc plans were reviewed and performed using the same methods as follows. The dynamic conformal arc technique is a forward‐planning method using multiple noncoplanar arcs rotating around a single isocenter, in which the MLC continuously changes its aperture to conform the beam to the target with every 5° of arc.^10^, ^11^ The MLC consists of two opposing banks of moveable tungsten leaves. Each banks is configured with 60 leaves, 120 leafs are moved along the some axis as the X jaws. The treatment planning system (TPS) used was the BrainSCAN (IPlan3.0) (BrainLAB) with 6 MV photon energy, MLC (5 mm/degree and 2.5 cm/s), dose rate (600 MU/min) and gantry speed $\left(72\;s/\text{turn}∼5^{\circ }/s\right)$ are optimized simultaneously to achieve the desired degree of modulation.

Volumetric kV CBCT data sets into the ExacTrac software and performs an automatic 6D fusion to the pretreatment CT images. The remote controlled treatment couch and robotic module allow for any shifts to be detected and compensated for from outside the treatment room. Afterwards, any patient motion is monitored in real time based on infrared markers attached to the patient's body. The ExacTrac X‐Ray 6D system has the ability for pretreatment positioning and intrafraction verification (snap mode).

Stereotactically localized CT scans were obtained with contiguous 3 mm slice. The CTV was defined matching CT and MRI studies. The CTV was expanded to a PTV with a 3 mm isotropic margin considering the practical setup uncertainty.

The number of arcs per plan used was five for a PTV in different table positions and arc lengths (i.e., the range between the start and stop angles of the gantry), and the collimator angle in all arcs was set at 0°. All treatment plans were normalized at the geometric isocenter of the CTV

#### B.1 Patient immobilization and initial setup

Patient immobilization was achieved by using the commercially available BrainLAB head mask fixation system. The characteristics of this system have been previously described.^(12)^

During the planning visit, a thermoplastic mask was fabricated to conform precisely to the patient. The mask incorporated three nonperforated thermoplastic reinforcing straps over the forehead, below the nose, and over the chin. Infrared fiducial reflectors were attached to the mask to assist in isocenter localization and monitoring of the patient's position.

The frameless compensates for the slight loss of immobilization by reducing other potential errors accumulating at each step in the radiotherapy procedure. Rather than manually driving the couch and positioning the patient indirectly by matching surface marks with laser crosshairs, an infrared camera system was implemented to maneuver the patient in the field of view of the image‐guided system to initiate the positioning process based on visualization of the internal anatomy. Dedicated software subsequently calculates the three‐dimensional deviations from the expected target position and corrects them by moving and rotating the couch on which the patient is immobilized in six directions.

Immediately before treatment, all patients underwent CT verification to check the accuracy of isocenter position with the infrared system. Firstly, the CT verification set was imported in the planning system and localized automatically by the planning software through identification of the stereotactic fiducials in the same way as for planning CT. Since this step spatially coregistered the stereotactic coordinate systems of planning CT and verification CT with the respect to the localizer box, errors in patient repositioning resulted in a shift of anatomical isocenter position. In the second step, the planning CT and the CT verification were fused. Following fusion, anatomy was coregistered. Since all brain structures were spatially matched, any translation of isocenter position due to patient repositioning error resulted in a mismatch of the localizer rods of the localizer box. As consequence, the 3D stereotactic coordinates of isocenter in the verification CT changed accordingly. Finally, the new isocenter coordinates were recorded, and the isocenter shift between verification and planning CT was calculated.

Positioning corrections were applied by automatic couch shifts in the longitudinal, vertical, and lateral directions; also roll, pitch, and yaw rotations were applied by automatic adjustment of the treatment angles. Consequently, image registration and patient setup corrections were made for six degrees of freedom.

#### B.2 Analysis

**Figure 2:**
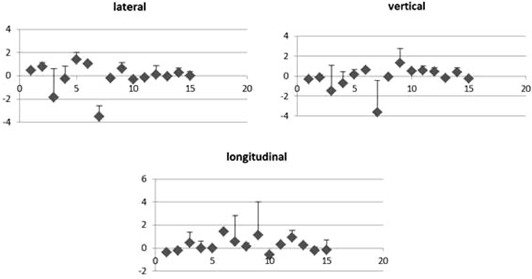
Patient positioning corrections mean (mm), error bars indicating standard deviations of the mean.

The primary objectives were to quantify daily setup errors; the secondary objective was to use the data obtained to calculate CTV–PTV margins for RT Figure 2 shows setup errors (translation and rotation) after correction, established with acquired X‐ray.

The 3D deviations are expressed as mean±standard deviation. The random error $\left(\Sigma (\sigma_{i})\right)$, systematic error $\left(\sigma_{i}\right)$, and group systematic error $\left(M(\mu_{i})\right)$ for the different X‐rays were calculated using the definitions of van‐Herk.^(1)^ Systematic error can be defined as the mean $\left(\mu_{i}\right)$ of positioning corrections for that patient, and random error can be defined as the standard deviation $\left(\sigma_{i}\right)$.^13^, ^14^ We termed global systematic error (mean of the systematic error distribution), patient‐to‐patient variation in systematic errors (standard deviation of the systematic error distribution), and magnitude of patient‐specific random errors (root mean square [RMS] of the random error distribution). The overall global systematic error, or the group systematic error, is defined as $M(\mu_{i})$, which is calculated by Eq. (1):

(1) $$M(\mu_{i})=\sum_{i=1}^{P}\frac{N_{i}\mu_{i}}{N}\;where\;\mu_{i}=\sum_{k=1}^{Ni}\frac{m_{k}}{N_{i}}$$

where $\mu_{i}$ is the individual systematic error for patient i, *P* is the total number of patients, *N* is the total number of treatment fractions, *N_i_* is the number of treatment fractions for patient i, and $m_{k}$ is the measured positioning correction for the kth fraction for patient i. Equation (2) gives the variation in systematic errors:

(2) $$\sum (\mu_{i})=\sqrt{\frac{\sum_{i=1}^{P}N_{i}{\left(\mu_{i}-M(\mu_{i})\right)}^{2}}{\left(N-1\right)}}$$

RMS$\left(\sigma_{i}\right)$ is the RMS of the random error distribution, which we defined as the magnitude of random errors. Equation (3) calculates the magnitude of random errors:

(3) $$RMS(\sigma_{i})=\sqrt{\sum_{i=1}^{P}\frac{(N_{i}-1){\left(\sigma_{i}\right)}^{2}}{\left(N-1\right)}}\;where\;\sigma_{i}=\sqrt{\frac{\sum_{k=1}^{N_{i}}{\left(m_{k}-\mu_{i}\right)}^{2}}{\left(N_{i}-1\right)}}$$

where $\sigma_{i}$ is the individual random error for patient i. The systematic and random error distributions are related to the overall distribution of setup corrections by Eq. (4):

(4) $$\sum_{overall}=\sqrt{\sum^{2}(\mu_{i})+RMS^{2}\sigma_{i}}$$

This equation indicates that the overall variation in positioning corrections is composed of the standard deviation of the systematic error distribution and the RMS of the random error distribution.^(1)^

The systematic error and random error were used to derive CTV‐to‐PTV setup margin, according to the margin recipes suggested by Stroom et al.^(15)^ or Van Herk et al.^(16)^

To analyze the effect of the setup error correction on the first day, we calculated and compared the planning margins required to compensate for setup errors. We used two margin recipes. Margin 1 is based on the formula from van Herk, ^(16)^ in which

(5) $$\begin{array}{l} \text{margin}\;1=\sqrt{2.7^{2}\sum^{2}+1.6^{2}\sigma^{2}-2.8} \end{array}$$

and margin 2 is based on the recipe from Stroom et al.,^(2)^ in which

(6) $$\text{margin}\;2=2.0*\sum (\mu_{i})+0.7*RMS\sigma_{i}$$

## III. RESULTS

The research was performed on 15 patients in whom altogether 45 measurements were made for evaluating the position of a patient during irradiation. For each patient, three to a maximum of five positioning measurements were carried out. A total of 225 X‐ray scans was obtained for the 15 patients; all allowed confident assessment of reference bony anatomy, providing an excellent setup error evaluation using automated image registration. Mean setup errors were calculated from X‐ray images acquired after all three fractions.

2, 3 show setup errors after correction, established with acquired plotted X‐ray and registration. The errors in all directions were comparable. Translation and rotational errors were small.

Variations in systematic errors (M(μi)) and magnitudes of random errors $\left(\text{RMS}(\sigma_{i})\right)$ are listed in Table 1.

The systematic error for the entire group in the lateral, craniocaudal, and anteroposterior axis was 1.21 mm, 1.89 mm, and 1.59 mm, respectively.

There is moderate patient‐to‐patient variation in the vertical direction, and small variations in systematic errors and magnitudes of random errors are smaller. The global systematic errors were measured to be less than 2.0 mm in each direction. Random component of all patients are smaller, ranging from 0.1–0.3 mm. The use of the same type of immobilization device (e.g., thermoplastic mask) and the rigid anatomy in the intracranial group may contribute to the similar setup uncertainties for brain patients.

**Figure 3:**
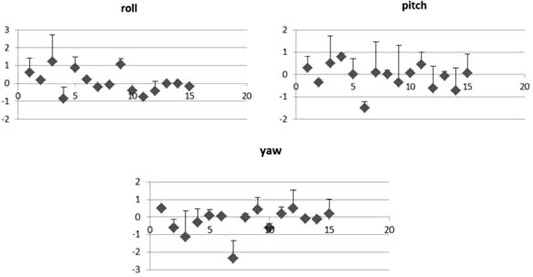
Patient rotational corrections mean (°), error bars indicating standard deviations of the mean.

$M(\mu_{i})$ and RMS$\left(\sigma_{i}\right)$ are typically used to describe the systematic and random errors. $M(\mu_{i})$ is overall global systematic error, and RMS$\left(\sigma_{i}\right)$ measures the variations of setup among different treatment fractions. Ideally, they should be at the same magnitude (i.e., variations of setup error between different patients and between different fractions for the same patient should be similar). In our case, the $M(\mu_{i})$ is much larger than RMS$\left(\sigma_{i}\right)$, indicating that the magnitude of the systematic error is larger than those of the random errors.

For almost all regions, the clinically applied isotropic margin of 3 mm was correct. The safety margin (SM), that would, with a 90% probability, cover an inaccurate positioning of a patient with respect to the lateral, is 0.5 mm and 2.6 mm for van Herk^(1)^ and Stroom et al.,^(2)^ respectively, craniocaudal axis is 1.5 mm and 3.4 mm, respectively, and with respect to the anteroposterior axis, 2.3 mm and 3.9 mm (Table 2).

There is a statistically significant difference between the two margins estimate (using two different margin recipes) (p=0.02); however, note that our institutional margin value of 3 mm lies inside the confidence interval of both recipes.

**Table 1:** Mean and standard deviation of setup variations in lateral (LR), longitudinal (CC), vertical (AP), and rotational directions

	*Systematic errors*		*Random errors*	
	*Mean (mm)*	*SD*	*Mean (mm)*	*SD*
Translational (mm)				
Lateral‐LR	1.21	1.33	0.20	0.49
Vertical‐AP	1.89	2.30	0.18	0.59
Longitudinal‐CC	1.59	1.73	0.27	1.09
Rotational (degrees)				
Roll	1.05	1.26	0.21	0.43
Pitch	1.60	1.52	0.25	0.59
Yaw	1.80	1.95	0.17	0.50

**Table 2:** Calculated margins (mm) based on margin recipes by Van Herk equation (Margin 1) and Stroom equation (Margin 2)

	*Margin 1*	*Margin 2*
Lateral‐LR	0.5	2.6
Vertical‐AP	2.3	3.9
Longitudinal‐CC	1.5	3.4
Roll	0.1	2.3
Pitch	1.5	3.4
Yaw	2.1	3.7

## IV. DISCUSSION & CONCLUSIONS

The aim of this study was to determine an adequate safety margin that would allow an acceptable exposure of a target area to irradiation.

With daily couch shifts, uncorrected rotations around the longitudinal axis are responsible for a significant residual patient setup error.

The margin can be appropriate by correcting couch rotations using a commercial 6D repositioning device. Additionally, the rotational corrections for all treatment sites were relatively small, with a maximum overall systematic error of 0.2° and a maximum variation of setup error of 1.8°. Results of setup error analysis are due in part to restricting specific degrees of freedom during image registration. If a yaw offset exists, when restricting yaw and pitch rotations during fusion, the restriction will increase the lateral shift correction. If a pitch offset exists, the restriction will increase the vertical and longitudinal shift correction. This is relevant for couch sag discrepancies, which will cause pitch offsets.

In addition to patient‐specific variables, setup errors are dependent on many institution‐specific variables. Various components of the entire treatment process will affect measured setup errors. External fiducials type and placement location, as well as positioning laser accuracy, can affect initial patient setup. If the positioning lasers are not accurately lined up to patient fiducials at the simulation room, in the planning station, and at the treatment room, measured setup errors will occur. Overall procedures governing the use of the image guidance system will affect measurements. The objects being aligned during image registration — for example, bony anatomy as opposed to tumor — will affect resultant setup corrections. If different lengths and portions of the target are imaged on different days, this could have an effect on the size and variation of setup corrections due to the inclusion or exclusion of certain anatomic features. An additional factor is human behavior. Interobserver variation in the interpretation of daily images will affect the registration process. Differences in overall anatomic knowledge, along with the technique (automatic vs. manual) and time spent on image fusion, could likely affect resultant setup corrections. In addition, setup errors depend on how well the patient was positioned before imaging, which depends on the training, skill level, and attention of staff members. Care should be taken to ensure the most accurate patient setup and image registration possible.

If IGRT is not available, Stroom et al.^(15)^ suggested a margin formula to deliver a full dose to the tumor. The equation suggested is Eq. (6), where Σ is systematic error and σ is random error. This margin is intended to cover 99% of CTV volume with 95% of the prescribed dose. Using the equation by Stroom et al., we calculated the margins in Table 2. The suggested margin ranged from 2.6 (mean) mm, 3.4 mm, and 3.9 mm, for lateral, longitudinal, and vertical directions, respectively. Our results were comparable to other studies in the literature evaluating setup variations. For example, the setup variations for brain were reported between 2–5 mm (lateral), 1–5 mm (longitudinal), and 1–6 mm (vertical).^(16)^ It is not our aim to verify the equations proposed by Stroom et al.^(2)^ or Van Herk,^(1)^ because we believe that the margin should be determined by multiple factors, including treatment goals, tumor stages, tumor/normal tissue locations, immobilization technique, and confidence level. The margin formulas may then be used only for confirmation. In the margin recipes proposed, setup error and organ motion uncertainties were the only parameters included in the equation. In reality, CTV delineation uncertainties exist and should be included in the CTV for a more accurate margin to a better tumor control. Weiss and Hess^(17)^ suggested that interobserver variability in target delineation was a major factor causing treatment inaccuracy. The delineation uncertainty can be reduced by using multimodality imaging as suggested by Rasch et al.^(18)^ The overall CTV‐to‐PTV margin, including tumor delineation, setup uncertainties, and internal organ displacement, should be fully investigated before determining PTV margin. In addition, IGRT minimizes the interfractional setup errors, and the margin derived from this study should not be used if IGRT is available.

The ExacTrac X‐Ray 6D system uses kV X‐ray to obtain 2D localization images with high spatial and contrast resolutions. The 6D fusion algorithm provides optimal match between the 2D localization images and the 3D CT simulation images. The infrared‐based ExacTrac system provides precise control of patient positions and makes the accurate online adjustment of the patient position possible. In addition, the X‐ray system is in fixed positions so that its isocenter is fixed and consistent with the linac isocenter defined by the room laser system. This makes it an excellent IGRT system for targets attached to internal rigid bony structures, such as cranial and spinal lesions. The radiation delivered to the patient during imaging is negligible compared to cone‐beam CT or 2D MV portal images.

The present study emphasizes the importance of daily three‐dimensional imaging in all conformal and IGRT radiation therapy. Daily X‐ray imaging is essential to compare and assess the accuracy of treatment delivery to different anatomical locations. This study also emphasizes the importance of monitoring setup error for all treatment fractions.

## Supporting information

Supplementary Material
