# Supplementary material for: Estimation of patient setup uncertainty using BrainLAB Exatrac X‐Ray 6D system in image‐guided radiotherapy
Source: J Appl Clin Med Phys. 2015 Mar 8;16(2):99–107. doi: 10.1120/jacmp.v16i2.5102 (PMC5690103; doi:10.1120/jacmp.v16i2.5102)
Supplement: Supplementary file 1 — Supplementary Material [file ACM2-16-099-s001.doc]

Referee remarks:
This paper studied the estimation of patient setup uncertainty using
brainlab Exactrac X-ray 6D system in image-guided radiotherapy. This paper
has clinically significance. It is recommended to consider publishing this
paper. However, there were multiple typos and some sentences were not very
clear.

Line 6-8: “ExacTrac X-Ray 6D system was used to verify patient position
and tumor target localization before each treatment, setup variations
(translation and rotation) after correction were recorded and corrected
before treatment.”
Before treatment, did correction based on cone-beam CT? If it was, it is
better to mention cone-beam CT here.
“setup variations (translation and rotation) after correction were
recorded and corrected before treatment” was not very clear, it is hard to
understand author’s meaning here.

the correction is not based on CBCT, bat on two x-ray images with the ExacTrac (infrared) system. The 6D fusion software first generates various sets of DRRs with position variations in both 3 translational and 3 rotational directions (6 degrees of freedom) for the CT images.

I changed the text

Line 44-47: “This study analyzes patient positioning corrections that are
performed during treatments using an ExacTrac X-Ray 6D system, that is
mainly an integration of 2 subsystems: an infrared (IR)-based optical
positioning system (ExacTrac) for initial patient setup and precise control
of couch movement, and a radiographic kV x-ray imaging system (X-Ray 6D) for
position verification and readjustment.”
During treatments, how to do the correction and how to do the readjustment?

The two x-ray images and the 6D fusion software that generates the sets of DRRs with position variations in translation and rotational directions was used before any treatment fields and adjusted during treatments

I added it to the text

Line 73-75: “The 6D fusion software first generates various sets of DRRs
with position variations in both 3 translational and 3 rotational directions
(6 degrees of freedom) for the CT images.”
Don’t understand this sentence. Was “for” “from”? Please specify
“CT images” as CBCT images or planning CT images.

correct

Line 94-97: “Volumetric kV CBCT data sets into the ExacTrac software and
performs an automatic 6D fusion to the pre-treatment CT images. The remote
controlled treatment couch and robotic module allow for any shifts to be
detected and compensated for from outside the treatment room. After wards
any patient motion is monitored in real-time based on infrared markers
attached to the patient’s body.”
Patient motion was monitored in real-time. Did snap mode use? Was there a
tolerance of position changing for readjustment?

the system use snap mode and we always change position for readjustment

Line 98 – 99: “The CTV was defined matching CT an MRI studies.”
Please correct this sentence.

correct

Line 79, “Between October 2014 and March 2014”
“October 2014” was not correct.

correct, I reversed the dates

Line 110: “raditherapy”
Please correct.

correct

Line 116, “Immediately before treatment, all patients underwent CT
verification to check the accuracy of isocenter position.”
Did the “CT verification” means CBCT?

CT verification that checks the accuracy of isocenter position is obtained with the infrared system
correct the text

Line 129: “The primary objectives were to quantify daily setup errors;”
The setup errors were recorded and analysed in this study. Was any
reposition performed if the setup error was higher than a value, such as 1
mm?

repositioning is performed for all values

Line 181
“Traslation and rotational errors were small” - Should be Translation

correct
